# What can we learn about acid-base transporters in cancer from studying somatic mutations in their genes?

**DOI:** 10.1007/s00424-023-02876-y

**Published:** 2023-11-24

**Authors:** Bobby White, Pawel Swietach

**Affiliations:** Department of Physiology, Anatomy and Genetics, University of Oxford, Parks Road, Oxford, OX1 3PT UK

**Keywords:** pH regulation, Acidosis, Solute-linked carrier, Somatic mutation, Cancer evolution, Glycolysis

## Abstract

Acidosis is a chemical signature of the tumour microenvironment that challenges intracellular pH homeostasis. The orchestrated activity of acid-base transporters of the solute-linked carrier (SLC) family is critical for removing the end-products of fermentative metabolism (lactate/H^+^) and maintaining a favourably alkaline cytoplasm. Given the critical role of pH homeostasis in enabling cellular activities, mutations in relevant SLC genes may impact the oncogenic process, emerging as negatively or positively selected, or as driver or passenger mutations. To address this, we performed a pan-cancer analysis of The Cancer Genome Atlas simple nucleotide variation data for acid/base-transporting SLCs (ABT-SLCs). Somatic mutation patterns of monocarboxylate transporters (MCTs) were consistent with their proposed essentiality in facilitating lactate/H^+^ efflux. Among all cancers, tumours of uterine corpus endometrial cancer carried more ABT-SLC somatic mutations than expected from median tumour mutation burden. Among these, somatic mutations in *SLC4A3* had features consistent with meaningful consequences on cellular fitness. Definitive evidence for ABT-SLCs as ‘cancer essential’ or ‘driver genes’ will have to consider microenvironmental context in genomic sequencing because bulk approaches are insensitive to pH heterogeneity within tumours. Moreover, genomic analyses must be validated with phenotypic outcomes (i.e. SLC-carried flux) to appreciate the opportunities for targeting acid-base transport in cancers.

**Supplementary Information:**

The online version contains supplementary material available at 10.1007/s00424-023-02876-y.

## Introduction

Solute-linked carriers (SLCs) are a superfamily of genes integral to physiological cellular function and wider homeostasis across organ systems. SLCs comprise 66 gene families that code for transporters of ions and solutes across biological membranes [39, 59]. This classification was introduced after a decade of intensive cloning, starting in the 1980s, that assigned genes to measurable fluxes across membranes [38, 45, 70]. There are at least 400 SLC members classed under broad groupings, such as transporters of bicarbonate (or carbonate [52]), monosaccharides, amino acids, and metal cations [39, 59]. SLC function is essential in the context of membrane transport because most ions and many polar solutes cannot freely diffuse across the phospholipid bilayer so require facilitation by proteins. Moreover, some SLCs can support active transport, which is often necessary for cellular homeostasis, substrate sequestration, waste excretion, and trans-epithelial transport. Illustrative of their biological importance, over 100 SLCs have been associated with human genetic disorders [72], and homozygous loss of certain SLCs produces embryonic lethality [84].

The acid/base-transporting SLCs (ABT-SLCs) play a crucial homeostatic role in facilitating the extrusion of acidic products of metabolism (notably lactate/H^+^) and maintaining a favourable intracellular pH (pHi) by balancing the import and export of H^+^-equivalents. Thus, ABT-SLCs can be grouped into ‘acid-loaders’ or ‘acid-extruders’, although the net direction of transport will depend on ionic gradients and regulatory cues [60]. In well-perfused normal tissues, extracellular pH (pHe) is tightly clamped at 7.4 by continuous capillary perfusion [11]. In contrast, tumour vasculature is chaotically organised and leaky, resulting in poor acid clearance [71]. pHe measurements in humans by specialised magnetic resonance imaging (MRI) modalities, like Chemical Exchange Saturation Transfer, put the median intra-tumoural pHe of various cancers, including breast cancer, hepatic carcinoma, prostate cancer, and glioma, at 6.8 [11]. Reported values reach as low as 6.3, and it is likely that the spatial resolution of MRI excludes the detection of microscopic pockets of more extreme acidity [11].

ABT-SLCs impinge upon homeostatic processes in cancer cells in three ways. Firstly, protons are a metabolic by-product, in the form of either lactate/H^+^ produced by fermentation or respiration-generated CO_2_ (which hydrates to HCO_3_^-^ and H^+^) [76]. Whereas CO_2_ can cross the lipid bilayer of membranes freely, lactate/H^+^ is poorly permeant without facilitation by SLC16-type proteins. Higher glycolytic rates are typically matched by higher SLC16-dependent membrane permeability to lactate/H^+^ [74]. This aids in preventing cytoplasmic acidification, which would otherwise exert negative feedback on glycolytic enzymes such as glyceraldehyde 3-phosphate dehydrogenase and phosphofructokinase 1 [61, 81, 83]. Secondly, it is imperative that cancer cells maintain a favourable pHi [60] as biological processes (with the exception of those compartmentalised to organelles of specific pH, like lysosomes or mitochondria) operate optimally around the mildly alkaline pH of 7.2 [60]. Outside the conducive pHi range, cancer cells are unable to engage in aggressive behaviours such as invasion and metastasis [11, 60]. Thirdly, secondary-active ABT-SLCs require considerable energetic input [27], especially considering the demand to maintain a relatively alkaline pHi in an acidic microenvironment (i.e. considerable uphill transport against an electrochemical gradient) [14, 60, 73, 92]. Moreover, many cancers develop from epithelia that transport large and complementary acid-base fluxes across apical and basolateral membranes as part of wider systems-level processes, such as acid secretion by the stomach or bicarbonate secretion by the exocrine pancreas. Such secondary active transport can carry a considerable energetic cost, despite no immediate survival benefit to the epithelial cell. Under finite resources, cancer cells must balance energetic flows to ABT-SLCs against other priorities, such as hyperproliferation [9].

The fundamental link between ABT-SLCs and cancer metabolism begs the question: do changes in acid-base transport influence tumourigenesis in patients? This question is especially pertinent because inhibitors of ABT-SLCs have been mooted as therapeutic targets in cancer [19, 37, 66] and some reached early-phase clinical trials [37]. Direct appraisal of in vivo ABT-SLC flux in human cancers is currently unfeasible. Nonetheless, genomic analysis of human tumours could inform about how ABT-SLCs impact cancer cell fitness in vivo and their ultimate role in intra-tumoural evolution, which arises from the vast genetic and epigenetic heterogeneity among cancer cells [10, 65]. Where somatic mutations in a specific gene augment or impair cancer cell fitness, positive or negative selection, respectively, ensue [56]. A cancer’s landscape of somatic point mutations, insertions, and deletions can be harnessed to interrogate selection events that have occurred over the course of tumourigenesis [6, 7, 28, 50, 56, 90]. Indeed, somatic mutation-based approaches have successfully identified many genes that can drive cancer [6, 7, 28, 50, 56] and processes that are essential for cancer cell survival in human tumours [7, 90]. However, the landscape of somatic mutations in ABT-SLCs across the common types of human cancers is in need of being documented systematically, with the major caveat that it is not intuitive to predict their functional outcomes in terms of transport, i.e. actual phenotype that determines fitness.

Here, we perform a pan-cancer analysis of somatic mutations in ABT-SLCs using human tumour datasets from The Cancer Genome Atlas (TCGA) [16]. In a comparative approach, whereby the mutation status of acid-base transporters is contextualised with that of other cancer-relevant SLC classes [39, 59], we evaluate the role of acid-base transport in intra-tumoural evolution. Specifically, we discuss (i) whether there is evidence that mutations in ABT-SLCs drive cancer; (ii) the essentiality of ABT-SLCs to cancer; and (iii) the degree of redundancy within the pHi regulatory mechanisms of cancer cells.

## Pan-cancer analysis of somatic mutations in ABT-SLCs

**Fig. 1:**
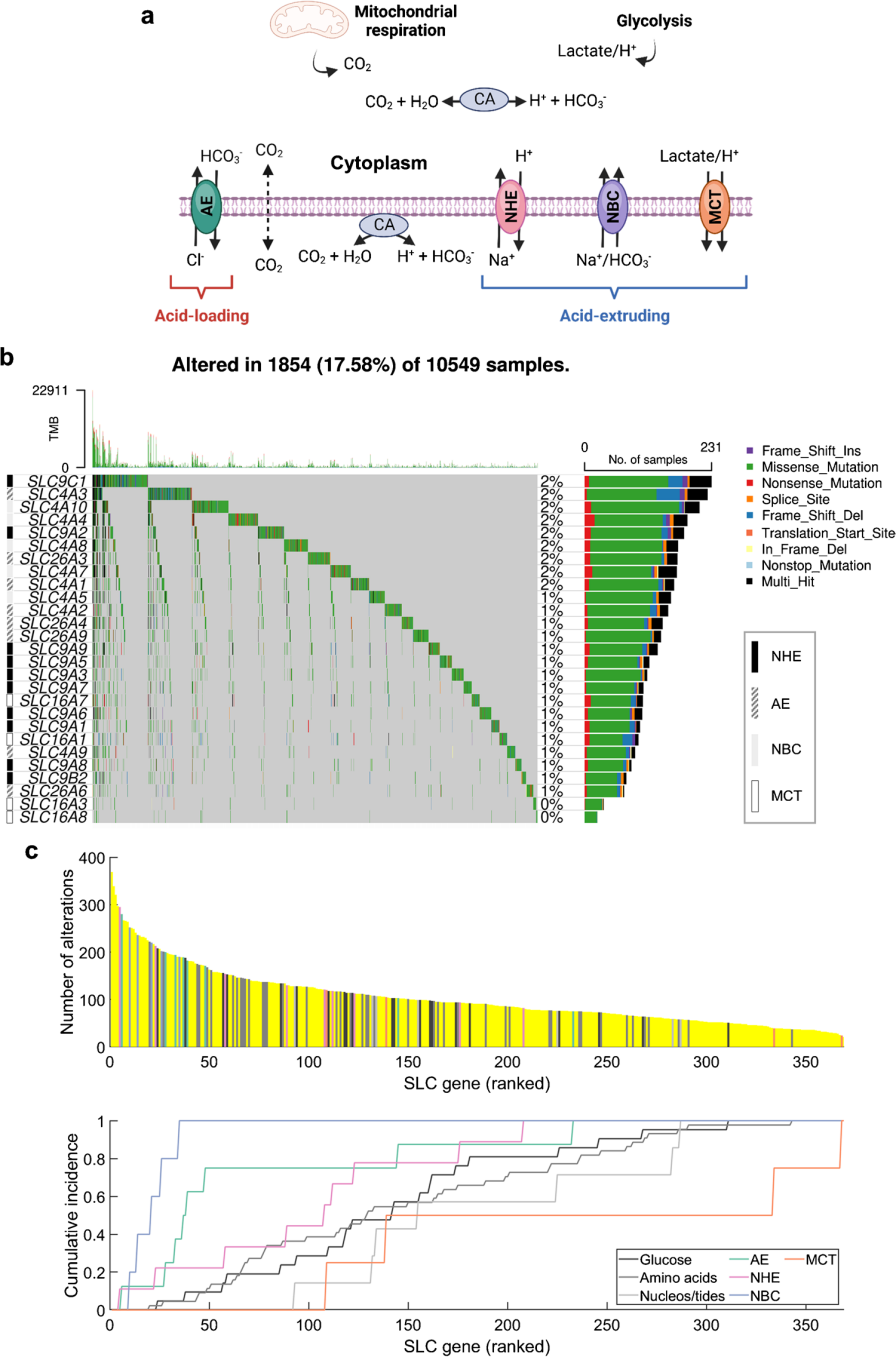
Pan-cancer analysis of simple nucleotide variation in ABT-SLCs. **a** Schematic of predicted ABT-SLC direction (excluding SLC4A5) at the plasma membrane of fermentive cancer cells under physiological conditions. Created with BioRender.com. **b** Open-access simple nucleotide variation data for all TCGA projects available for download from the GDC portal via TCGAbiolinks. Oncoplot of somatic mutations in ABT-SLCs. Percentages displayed are the number of samples carrying at least one somatic mutation in the given ABT-SLC normalised to the total number of samples analysed (*n*=10,549 samples). **c** Somatic mutation cumulative incidence for SLCs (ranked), highlighting groups of SLCs: all glucose-transporting SLCs, amino acid-transporting SLCs, nucleotide/nucleoside-transporting SLCs, AEs, NHEs, NBCs, and MCTs. The cumulative incidence plot illustrates the distribution of SLCs by grouping across the full range of SLCs, ranked by descending incidence of mutations

We subdivided ABT-SLCs into 4 groups based on the type of flux carried: monocarboxylate transporters (MCTs), sodium-hydrogen exchangers (NHEs), sodium-bicarbonate cotransporters (NBCs), and anion exchangers (AEs) [74]. We took a conservative approach and curated ABT-SLCs on the basis of a proven role in acid-base transport across the surface membrane, rather than sequence similarity to known ABT-SLCs [39, 59]. The classification of individual genes, their substrates, and transport type are summarised in Table 1. Under physiological scenarios, NHEs and most NBCs (with the notable exception of SLC4A5 [8]) are predicted to be acid-extruders, AEs are predicted to be acid-loaders, and in fermentive cancer cells, MCTs are predicted to be acid-extruders (Fig. 1a) [12, 74]. Importantly, the direction of transport is a function of numerous microenvironmental conditions, which may differ in tumours, namely: oxygen levels, pH, ATP levels, and lactate [9, 11]. The expression of these SLCs is recognised to be regulated by oncogenic pathways [21], nutrient-sensing mechanisms [60], and gene methylation [33]. Yet, there is little information about their somatic mutations in cancer. Somatic mutations in *SLC16A1* [18], *SLC16A7* [24, 64], *SLC9A2* [91], *SLC9A3* [42], *SLC9A8* [48], *SLC9A9* [31, 85], *SLC4A2* [22, 88], *SLC4A4* [53], *SLC4A7* [34], and *SLC4A8* [47] have been reported in human cancers (Table 1). However, their abundance across large pan-cancer cohorts has not been analysed. To address this gap, we downloaded open-access simple nucleotide variation data from all available TCGA projects on the GDC data portal [16] via TCGAbiolinks (R) [23]. The full list of TCGA cohorts included in analyses is denoted in Supplementary Table 1. Maftools (R) was then used to analyse and present the downloaded somatic mutation data [57].

**Table 1:** ABT-SLC functions and previously documented cancer-linked variants

Gene	Common aliases	Predicted acid-base transport in cancer cells (physiological conditions)	ABT-SLC group	Substrates	Transport type (ABT-SLC role)	Cancer-linked germline SNPs or somatic mutations
*SLC16A1*	MCT, MCT1, MCT1D, HHF7	Acid-extruder in fermentive cells	MCT	Acetoacetate, D-beta-hydroxybutyrate, ketone bodies, lactate, monocarboxylates, pyruvate, H^+^	Symport (H^+^/monocarboxylate)	Nonsense or missense somatic mutations at R328Q/* (pan-cancer) [18]
*SLC16A3*	MCT3, MCT4	Acid-extruder in fermentive cells	MCT	Acetate, acetoacetate, beta-hydroxybutyrate, ketone bodies, lactate, H^+^	Symport (H^+^/monocarboxylate)	N/A
*SLC16A7*	MCT2	Acid-extruder in fermentive cells	MCT	Ketone bodies, lactate, pyruvate, H^+^	Symport (H^+^/monocarboxylate)	Somatic amplification (metastatic osteosarcoma) [64]; rare ALK fusion partner (NSCLC) [24]; SNP rs995343 (NSCLC & CRC) [32, 35]
*SLC16A8*	MCT3, REMP	Acid-extruder in fermentive cells	MCT	Lactate, H^+^	Symport (H^+^/monocarboxylate)	N/A
*SLC4A1*	AE1, RTA1A, CD233, EPB3, Diego blood group	Acid-loader	AE	Cl^-^, HCO_3_^-^	Antiport (Cl^-^/HCO_3_^-^)	N/A
*SLC4A2*	AE2, BND3L, HKB3, EPB3L1, NBND3, MPB3L	Acid-loader	AE	HCO_3_^-^, Cl^-^	Antiport (Cl^-^/HCO_3_^-^)	Somatic mutations (renal cell carcinoma) [88]; copy number gain (prostate cancer) [22]
*SLC4A3*	AE3, SLC2C, CAE3/BAE3, SQT7	Acid-loader	AE	HCO_3_^-^, Cl^-^	Antiport (Cl^-^/HCO_3_^-^)	N/A
*SLC4A9*	AE4, SBC5		AE	Cl^-^, HCO_3_^-^	Antiport (Cl^-^/HCO_3_^-^)	N/A
*SLC26A3*	Chloride anion exchanger, DRA	Acid-loader	AE	HCO_3_^-^, Cl^-^	Antiport (Cl^-^/HCO_3_^-^)	N/A
*SLC26A4*	PDS, pendrin	Acid-loader	AE	Cl^-^, I^-^, HCO_3_^-^	Antiport (Cl^-^/HCO_3_^-^)	N/A
*SLC26A6*	Anion exchange transporter, sulfate anion transporter, anion transporter 1, pendrin L1	Acid-loader	AE	Cl^-^, oxalate, SO_4_^2-^, HCO_3_^-^	Antiport (HCO_3_^-^/Cl^-^)	N/A
*SLC26A9*	Anion transporter/exchanger protein 9	Acid-loader	AE	Cl^-^, oxalate, SO_4_^2-^, HCO_3_^-^	Antiport (HCO_3_^-^/Cl^-^)	N/A
*SLC4A4*	NBCe1, NBC, NBC1, NBC2, HNBC1, HhNMC, KNBC1, PNBC	Acid-extruder	NBC	HCO_3_^-^, Na^+^	Symport (Na^+^/HCO_3_^-^)	Novel focal amplifications (prostate cancer) [53]
*SLC4A5*	NBCe2, NBC4	Acid-loader	NBC	HCO_3_^-^, Na^+^	Symport (Na^+^/HCO_3_^-^)	N/A
*SLC4A7*	NBCn1, NBC2, NBC2B, NBC3, SLC4A6, SBC2, BT	Acid-extruder	NBC	HCO_3_^-^, Na^+^	Symport (Na^+^/HCO_3_^-^)	SNP rs4973768 (breast cancer) [20, 95]; recurrent mutation (refractory multiple myeloma) [34]
*SLC4A8*	NBC, NBC3, electroneutral sodium bicarbonate exchanger 1, electroneutral Na(+)-driven Cl-HCO3 exchanger, K-NBC3	Acid-extruder	NBC	HCO_3_^-^, Na^+^	Symport (Na^+^/HCO_3_^-^)	Somatic mutations (thyroid microcarcinoma) [47]
*SLC4A10*	Sodium-driven chloride bicarbonate exchanger, NBCn2, NCBE	Acid-extruder	NBC	Cl^-^, HCO_3_^-^, Na^+^, H^+^	Symport (Na^+^/HCO_3_^-^)	N/A
*SLC9A1*	NHE1, APNH, PPP1R143	Acid-extruder	NHE	H^+^, Li^+^, Na^+^	Antiport (Na^+^/H^+^)	N/A
*SLC9A2*	NHE2	Acid-extruder	NHE	H^+^, Na^+^	Antiport (Na^+^/H^+^)	Recurrent somatic synonymous mutations (melanoma) [91]
*SLC9A3*	NHE3	Acid-extruder	NHE	H^+^, Na^+^	Antiport (Na^+^/H^+^)	Frequent somatic co-mutation with KRAS (pan-cancer) [42]
*SLC9A5*	NHE5	Acid-extruder	NHE	H^+^, Na^+^	Antiport (Na^+^/H^+^)	N/A
*SLC9A6*	NHE6	Acid-extruder	NHE	H^+^, Na^+^	Antiport (Na^+^/H^+^)	N/A
*SLC9A7*	NHE7, SLC9A6	Acid-extruder	NHE	H^+^, K^+^, Na^+^	Antiport (Na^+^/H^+^)	N/A
*SLC9A8*	NHE8	Acid-extruder	NHE	H^+^, Na^+^	Antiport (Na^+^/H^+^)	MSI target gene (colorectal cancer) [48]
*SLC9A9*	NHE9	Acid-extruder	NHE	H+, K+, Na+	Antiport (Na^+^/H^+^)	Somatic mutations (acute myeloid leukaemia) [85]; somatic mutations (neuroblastoma) [31]
*SLC9B2*	NHA2, NHEDC2, NHE10	Acid-extruder	NHE	H^+^, Na^+^	Antiport (Na^+^/H^+^)	N/A
*SLC9C1*	SLC9A10, NHE, Sperm-NHE, SNHE	Acid-extruder	NHE	H^+^, Na^+^	Antiport (Na^+^/H^+^)	N/A

Strikingly, 17.58% (1,854/10,549) of tumour samples carried at least one somatic mutation in a gene coding for an ABT-SLC, yet individual ABT-SLC genes were somatically mutated in only 0–2% of tumour samples (Fig. 1b). The majority of mutations changed protein coding sequence (i.e. missense). The most commonly mutated ABT-SLC was *SLC9C1*, previously reported to be a sperm-specific NHE which, unlike the *SLC9A* family, is voltage-gated [87]. Overall, MCTs tended to be the least abundantly mutated ABT-SLCs (somatic mutations present in 0–1% of tumour samples), whereas NBCs were collectively some of the most commonly mutated (somatic mutations present in 1–2% of tumour samples).

We next sought to contextualise these findings against other SLC transporters (Fig. 1c) [39, 59]. Glucose uptake by SLCs is required to sustain the high glycolytic rate of cancer cells, a vital source of biosynthetic intermediates for cell proliferation [94]. Amino acids, uptake of which is SLC-mediated, are the building blocks of proteins and precursors to numerous metabolites essential for cellular function, including C1 compounds, nucleotides, glutathione, polyamines, hexosamines, and creatinine [15]. Another important SLC group is the nucleotide/nucleoside transporters which deliver bases of nucleic acids. In order to compare ABT-SLCs against glucose-, amino acid-, or nucleotide/nucleoside-transporting SLCs, we first ranked all somatically mutated SLC genes by descending mutation incidence (Fig. 1c, *top*). The cumulative incidence of somatic mutations along the SLC ranking was then calculated for each transport group (Fig. 1c, *bottom*).

In terms of the number of somatic alterations, glucose- and amino acid-transporting SLCs featured uniformly across the range of all SLCs, without enrichment among highly or lowly mutated genes (Fig. 1c). Nucleoside/nucleotide-transporting SLCs tended to have a below-average mutation incidence among SLCs. In terms of ABT-SLCs, NBCs and AEs generally had an above-average mutation incidence among SLCs, whereas MCTs had a below-average incidence (Fig. 1c). These observations indicate that the mutation rates among NBCs and AEs are relatively high among SLCs, whereas mutations in MCTs may be selected negatively. Specifically, the NHEs *SLC9C1* and *SLC9A2*, the NBCs *SLC4A10* and *SLC4A4*, and the AE *SLC4A3* ranked among the top 20 somatic SLC mutations most abundantly carried by tumour samples. In fact, *SLC9C2* mutations affected the highest proportion of tumour samples out of all SLCs, which is notable because, although it is currently an orphan transporter [59], its sequence is closely related to *SLC9C1*, the voltage-gated NHE [87]. When contextualised against all SLCs, the relatively high mutation incidence of certain ABT-SLCs warrants further investigation as to whether their mutations have undergone positive selection.

## Can mutations in acid-base transporter genes drive cancer?

Cancer driver genes are defined as genes whose mutations increase net cell growth under the specific microenvironmental conditions present in vivo and are estimated to comprise 1–3.9% of somatic mutations [56]. Importantly, cancer driver genes are the basis of targeted anti-cancer therapies. Extrapolating from in vitro findings, it could be speculated that gain-of-function mutations in acid-extruders support cell division by improving pHi homeostasis under intra-tumoural acidosis [4]. Moreover, the germline single nucleotide polymorphism (SNP) in the NBC *SLC4A7*, rs4973768, is associated with increased lifetime breast cancer risk, putatively due to *SLC4A7* overexpression [20, 95]. The germline SNP in the MCT *SLC16A7*, rs995343, has also been associated with adverse outcomes in colorectal and non-small cell lung cancers (Table 1) [32, 35]. Conversely, loss-of-function mutations in the Na^+^-coupled secondary active transporters, NBCs and NHEs, could divert ATP towards cell division programmes, particularly in cancers developing from tissues with substantial trans-epithelial solute movement [27]. Thus, there is good reasoning behind testing the notion of ABT-SLCs as cancer driver genes.

It is well-recognised that only *few* cancer driver genes are mutated in a high percentage of certain cancers [50]. Pertinent examples include *BRAF* in ~50% of melanomas and *PIK3CA* in ~25–30% of breast and colorectal cancers [5, 44, 46]. However, *most* cancer driver genes are mutated at intermediate rates, 2–20% of tumours [50], a threshold that is met by numerous ABT-SLCs pan-cancer (Fig. 1b). Cancer driver genes are typically identified by genomic methods in which two characteristics are assessed [28]: (i) whether their mutation frequency is in excess over background mutation rate and (ii) whether their mutations cluster at genomic loci corresponding to residues that are critical for protein function. These characteristics arise from positive selection as a consequence of the mutation’s survival benefit, relative to non-mutant cancer cells.

**Fig. 2:**
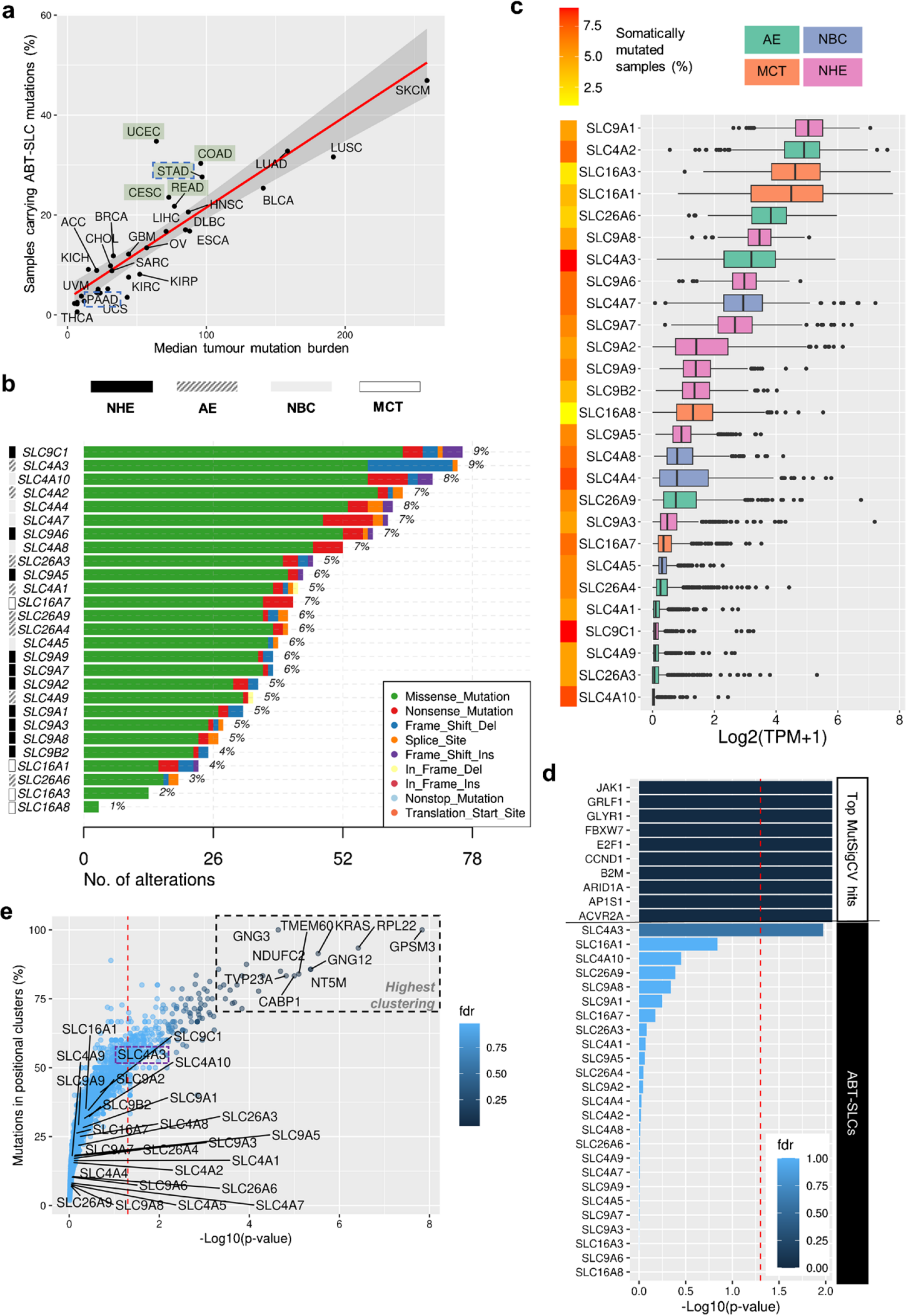
Characteristics of cancer driver genes in ABT-SLCs in UCEC. **a** Percentage of samples possessing at least one somatically mutated ABT-SLC plotted against the median number of somatic mutations per sample for each TCGA project. Linear model (red) fitted to data with 95% confidence interval (grey). **b**–**e** TCGA-UCEC project analysis. (**b**, **d**–**e**) *n*=518 samples. **b** Of all UCEC samples analysed, the percentage of samples carrying somatic mutations in ABT-SLCs, and the mutation type. **c** Log2(TPM+1) counts of ABT-SLC mRNA in primary tumours ranked by median. *n*=553 samples. **d**–**e** *p*=0.05 denoted by red dashed line. **d** MutSigCV analysis performed on the GenePattern server (http://cloud.genepattern.org) using default coverage and covariate files. Results displayed for ABT-SLCs and the 10 most significant (by *p*-value) genes with fill denoting fdr (*q*-value). **e** Proportion of somatic mutations clustering at genomic loci plotted against statistical significance as calculated by the oncodrive function in maftools. ABT-SLCs and top 10 most significant (by *p*-value) genes labelled

Unsurprisingly, we find that the cancer types most likely to carry somatic mutations in ABT-SLCs are known to have the highest background mutation rates (Fig. 2a, *abbreviations defined in Supplementary Table* *1*). These include skin cutaneous melanoma (SKCM) and lung adenocarcinoma (LUAD) or squamous cell carcinoma (LUSC). To identify specific cancers where ABT-SLCs are more likely to be under positive selection, we stratified the proportion of tumour samples carrying at least one ABT-SLC somatic mutation by TCGA cohort, then plotted against the cohort’s median tumour mutation burden with linear model fitting (Fig. 2a). For most cancer types, there was a positive linear relationship between median tumour mutation burden and the proportion of samples carrying mutations in ABT-SLCs. Indeed, it is estimated that 97–98% of somatic mutations in cancer are simply passengers (i.e. not sufficiently advantageous to be positively selected, nor sufficiently deleterious to be negatively selected) [56]. However, some cancer types had an enrichment in ABT-SLC mutations which was not directly proportional to their median tumour mutation burden. These included uterine corpus endometrial carcinoma (UCEC), colon adenocarcinoma (COAD), stomach adenocarcinoma (STAD), cervical squamous cell carcinoma (CESC), and rectal adenocarcinoma (READ) (Fig. 2a, *highlighted in green*).

We selected UCEC for further analyses, given that it was furthest away from the linear relationship. Strikingly, in UCEC, the percentage of tumour samples carrying ABT-SLC somatic mutations was substantially greater than the pan-cancer average: 1–9% (Fig. 2b). In line with our pan-cancer findings, somatic MCT mutations were generally the least common ABT-SLC mutations among UCEC tumour samples, whereas AEs, NBCs, and specific NHEs were the most common somatic ABT-SLC mutations in UCEC tumour samples. The functional impact of somatic mutations on cell fitness is likely to be greater for transporters that are responsible for a significant component of ion/solute flux. Accepting concerns about non-stoichiometric coupling between transcript and proteins levels, we used ABT-SLC expression levels in the UCEC cohort as a surrogate of transport activity (Fig. 2c). Open-access transcriptome profiling (STAR–Counts workflow) data for TCGA-UCEC primary tumours were downloaded from the GDC data portal via TCGAbiolinks [23]. To compare absolute expression levels between ABT-SLCs, transcripts per million (TPM) counts were analysed. Importantly, there was relatively high expression of the AEs *SLC4A2*, *SLC26A6*, and *SLC4A3*, and the MCTs *SLC16A3* and *SLC16A1*, which represent some of the most and least commonly somatically mutated ABT-SLCs in UCEC (Fig. 2b).

Whilst the analysis in Fig. 2a accounted for variation in median tumour mutation burden between cancer types, it is also important to consider that background mutation frequencies vary along an individual genome. This information is important when assessing whether a certain gene is mutated in excess over its expected background mutation rate [50]. To this end, we employed the MutSigCV algorithm to identify cancer driver genes in our UCEC simple nucleotide variation data (Fig. 2d) [51]. MutSigCV is considered a robust computational method because it accounts for multiple patient- and genomic position-based factors which can influence background mutation rate, including overall mutation rate and spectrum, DNA replication timing, and chromatin state estimation [51]. MutSigCV analysis was performed on the GenePattern server (http://cloud.genepattern.org) using default coverage and covariate files. None of the ABT-SLCs had a false discovery rate (fdr) below 5%, but *SLC4A3* had a significant non-adjusted *p*-value (*p*=0.0167) and emerged as an outlier to other ABT-SLCs (Fig. 2d).

We next sought to explore whether any ABT-SLCs expressed in UCEC fulfil the second characteristic of cancer driver genes: clustering around genomic loci corresponding to critical amino acid residues [28]. The oncodrive function (maftools R package) [57] is based on the OncodriveCLUST algorithm, which identifies genes with a significant bias towards mutational hotspots within the protein sequence [77]. We applied oncodrive to our UCEC simple nucleotide variation data. Overall, there was no evidence for significant clustering of mutations into specific functional domains in ABT-SLCs (Fig. 2e) [57]. However, it is notable that *SLC4A3*, which emerges as a highly mutated ABT-SLC, had the highest percentage of somatic mutations located in positional clusters (50.76%) among all ABT-SLCs (Fig. 2e, *purple dashed outline*).

**Fig. 3:**
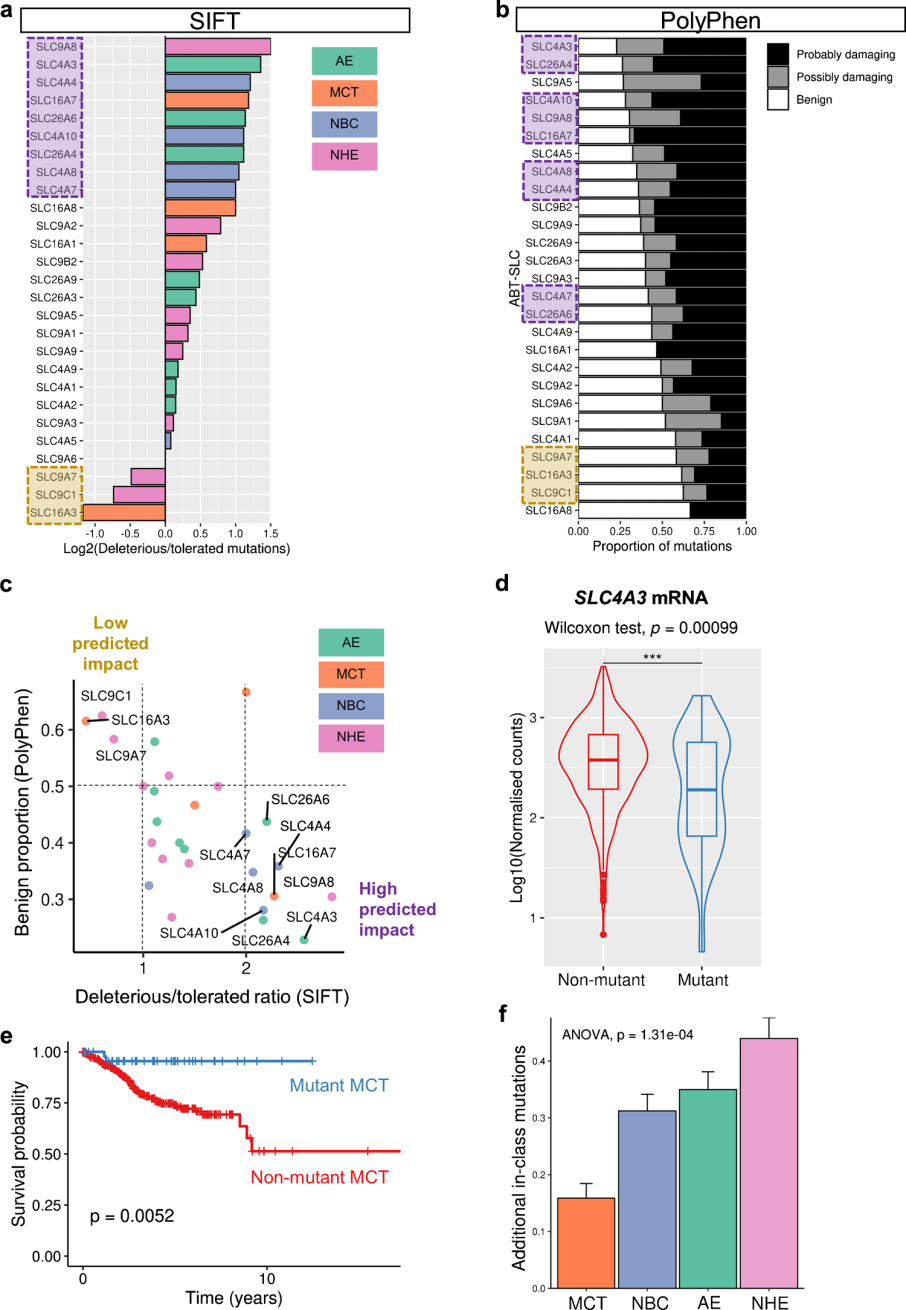
Functional consequences of somatic ABT-SLC mutations. **a**–**e** TCGA-UCEC project analysis. **a, b** *n*=154 samples. **a** Log2 of the ratio of somatic missense mutations predicted to be deleterious/tolerated by SIFT score for each ABT-SLC. **b** Proportion of somatic missense mutations denoted as benign, possibly damaging or probably damaging by PolyPhen-2 score for each ABT-SLC. **c** Ratios calculated in **a** plotted against the proportion of somatic missense mutations denoted as benign as calculated in **b**. Genes with <0.5 benign proportion and >2 deleterious/tolerated ratio labelled ‘high predicted impact’. Genes with >0.5 benign proportion and <1 deleterious/tolerated ratio labelled ‘low predicted impact’. **d** DESeq2-normalised (design ~1) *SLC4A3* mRNA counts for individuals with known *SLC4A3* somatic mutation status. Wilcoxon rank sum test. *n*=451 (non-mutant), 58 (mutant). **e** Overall survival analysis for TCGA-UCEC samples with both clinical and simple nucleotide variation open-access data available via TCGAbiolinks. *n*=461 (non-mutant), 49 (mutant in either *SLC16A1*, *SLC16A3*, *SLC16A7*, or *SLC16A8*). Log-rank test. **f** Pan-cancer analysis. Mean number of additional mutations/tumour in each ABT-SLC sub-group, in tumours carrying at least one mutation in that sub-group. *n=*729 (AE), 227 (MCT), 679 (NBC), 869 (NHE) tumour samples. One-way ANOVA

In addition to driver gene criteria based on mutation rate and positional clustering, it is pertinent to evaluate the predicted consequences of somatic mutations on protein function when considering positive selection. Indeed, only somatic mutations which alter cellular function may confer a differential survival advantage. To this end, we explored the PolyPhen-2 [1] and SIFT [63] scores included in the TCGA-UCEC simple nucleotide variation data download (Fig. 3a–c). PolyPhen-2 and SIFT predict the effects on protein function of missense mutations, which account for the vast majority of somatic ABT-SLC mutations in UCEC (Fig. 2b). SIFT predicted an excess of deleterious over tolerated mutations in the case of most ABT-SLCs, except for *SLC9A7*, *SLC9C1*, and *SLC16A3* (Fig. 3a). For most ABT-SLCs, fewer than half of mutations had benign effects on protein function as predicted by PolyPhen-2, except for *SLC9A1*, *SLC4A1*, *SLC9A7*, *SLC16A3*, *SLC9C1*, and *SLC16A8* (Fig. 3b).

To improve the accuracy of the functional predictions, we combined both SIFT and PolyPhen-2 to highlight ABT-SLCs whose mutations are predicted to have high and low impacts on protein function according to both approaches (Fig. 3c). Notably, *SLC9C1* mutations were predicted to have low functional impact, thus likely represent passenger mutations, yet *SLC9C1* was the most abundantly mutated ABT-SLC pan-cancer (Fig. 1b) and in UCEC (Fig. 2b). These observations may be explained by the low relative expression of *SLC9C1* (Fig. 2c), which suggests that SLC9C1 contribution to ensemble plasma membrane acid-base transport is minimal. Consequently, it is unlikely that *SLC9C1* mutations sufficiently alter acid-base transport to provide a survival advantage, from which positive selection for *SLC9C1* mutations with high functional impact could ensue. Strikingly, *SLC4A3* had the second highest ratio of deleterious/tolerated mutations (SIFT) and the highest proportion of non-benign mutations (PolyPhen-2). Given that we highlighted *SLC4A3* as having a high mutation burden, meaningful transcript levels, and a MutSigCV *p*-value <0.05, it is plausible that the enrichment of functionally damaging *SLC4A3* mutations may indicate a degree of positive selection [56].

To further explore the functional impact of *SLC4A3* somatic mutations, we analysed *SLC4A3* expression in tumour samples included in both our transcriptomic and simple nucleotide variation UCEC data. Lowly expressed genes were filtered out of the unstranded counts prior to DESeq2 normalisation [55]. Tumours carrying somatic *SLC4A3* mutations expressed significantly less *SLC4A3* mRNA (Fig. 3d). Interestingly, it has recently been shown that lysosomal degradation of its closely related isoform, SLC4A2, is an adaptation mechanism to low pHe in colorectal cancer cells which provides a relatively alkaline cytoplasm [60]. Consequently, we speculate that loss-of-function mutations to *SLC4A3* impair its acid-loading function in UCEC. The higher pHi attained this way would improve UCEC cell fitness under selection by intra-tumoural acidosis.

Previous large-scale pan-cancer algorithmic analyses have provided conflicting evidence as to whether ABT-SLCs can be cancer drivers [6, 28, 50, 56]. One of the first applications of algorithm-based detection to whole-genome pan-cancer data identified the AE *SLC26A3* and the NBC *SLC4A5* as drivers in 2–3% of glioblastoma multiforme and 3–5% of LUAD cases, respectively [50]. However, alternative algorithmic approaches have not confirmed any ABT-SLCs as cancer drivers [6, 28, 56]. These include a pan-software approach, where 26 different computational tools were used to validate candidate genes, and algorithms accounting for the differences in the typical nucleotide sequences flanking driver versus passenger mutations.

Cancer driver mutations are expected to confer an unambiguous advantage to cancer cells. The absence of ABT-SLCs among putative cancer driver genes identified in many analyses [6, 28, 56] may indicate that their mutations do necessarily confer an unequivocal fitness benefit. In carcinomas developing from epithelia, somatic mutations impairing trans-epithelial ABT-SLC-dependent transport may release ATP for hyperproliferation [27], which would benefit the cancer cell. For example, stomach adenocarcinoma (STAD) and pancreatic adenocarcinoma (PAAD) (Fig. 2a, *blue dash outline*) develop from epithelia that transport large and complementary acid-base fluxes across apical and basolateral membranes as part of wider systems-level processes. However, loss-of-function mutations in ABT-SLCs may cause weaker pHi control and lower steady-state pHi [4], unless compensated for by tandem loss-of-function in acid-loading transport (e.g. AE), a scenario that is unlikely to occur by chance alone. Thus, the overall fitness benefit of loss-of-function mutations in NBC and NHE genes is unclear, as this has to balance the greater availability of ATP against weaker pHi control. The converse would also be true for gain-of-function mutations, and the overall benefit to cancer cells may be conditional.

A second factor underpinning the uncertainty surrounding ABT-SLCs as cancer driver genes relates to spatial considerations in bulk whole-genome/exome sequencing analyses. Whether or not a somatic mutation provides a survival benefit to a cell, relative to neighbouring cells, is dependent upon selection pressures within its microenvironment [11]. pHe is spatially heterogenous within a tumour [69], and acidosis is more common at the invasive edge or central hypoxic core. Gain-of-function mutations in acid-extruders or loss-of-function mutations in acid-loaders, for example, might only provide a survival benefit in specific tumour regions. When cancer cells from acidic and non-acidic tumour regions are pooled for sequencing, acidosis-specific cancer driver genes could be obscured.

## Are genes coding for acid-base transporters essential to cancer?

Whereas cancer driver genes provide a *relative* survival benefit when mutated, essential genes are required in their wildtype form for the *absolute* survival of cancer cells [89]. Within a cancer, loss-of-function somatic mutations in essential genes can be negatively selected against. Whilst the direction of evolution in cancer is dominated by positive selection (1–3.9% of somatic mutations), it is estimated that 0.02–0.5% of somatic mutations do undergo negative selection [56]. Over the last decade, the development of CRISPR knockout screens has enabled the detection of genes essential for fitness in cancer cells in vitro [61, 89].

Notably, many therapeutics targeting essential genes are associated with limiting toxicities and have often failed Phase 2/3 clinical trials [17]. These include inhibitors of cell cycle controllers, epigenetic regulators, protein homeostasis, and DNA-damage responses. These failures are because many essential genes are common to both cancer cells and healthy tissues. A potential solution is to target pathways that are essential to cell survival only under microenvironmental conditions that are cancer-specific. Intra-tumoural acidosis is one such cancer-specific selection pressure [11]. Given that ABT-SLCs are required to maintain an alkaline pHi in cancer cells under low pHe [4], it is plausible that certain ABT-SLCs could be cancer-specific essential genes and therefore important therapeutic candidates. For example, *SLC9A1* genetic ablation may not inhibit cell line growth under control conditions [4], yet significantly impairs cancer cell survival at low pHe [61].

Whilst ABT-SLCs can be essential in vitro, it is critical to determine whether these findings translate to cancer patients. One such approach is to assess negative selection of somatic mutations in ABT-SLCs. Indeed, if somatic mutations in a particular gene were negatively selected, it would implicate that its loss compromises cell fitness, thus identifying essential genes in a patient’s cancer [7]. Intriguingly, somatic mutations in multiple SLC genes are thought to be negatively selected in cancer. In an analysis of 7546 individual tumour exomes from the TCGA database, negatively selected genes were found to be enriched for the transport of glucose, bile salts, organic acids, metal ions, and amine compounds [90]. Out of the negatively selected genes related to molecular transport, the most significant involved glucose transport and metabolism, including genes encoding the glycolysis enzyme glucokinase (*GCK*), the glucose importers GLUT1 (*SLC2A1*) and GLUT8 (*SLC2A8*), and MCT4 (*SLC16A3*). In a similar pan-cancer analysis, Bányai et al. identified *SLC2A1*, *SLC16A3*, and the glycolysis gene *G6PD* to be negatively selected in human cancers [7]. Clearly, the patterns of negative selection in human tumours paint a pro-glycolytic picture in which transport of the end-products, lactate/H^+^, by MCTs is essential for cancer cell fitness.

Our analyses are consistent with the notion that plasma membrane transport of substrates and products of fermentation is essential in human tumours. Pan-cancer, a relatively small proportion of tumours carried somatic mutations in MCTs (Fig. 1b-c). Moreover, in UCEC, a tumour cohort with sufficiently frequent MCT mutations to power such analyses, somatic MCT mutations were associated with significantly better overall survival (Fig. 3e). This finding implies that loss of wildtype MCTs could be detrimental to UCEC progression, a feature expected of an essential gene in cancer.

A mechanistic explanation for the proposed essentiality of MCTs in cancer is that their loss-of-function suppresses glycolytic rate [79] and compromises the supply of ATP and key biosynthetic intermediates for proliferation [94]. An alternative explanation may relate to the excessive build-up of intracellular lactate/H^+^ that could be deleterious to cell growth. Indeed, knock-down of *SLC16A3* expression in breast cancer cells reduces the capacity of pHi to recover from an acid load [4]. Pharmacological inhibition or genetic silencing of *SLC16A1* and/or *SLC16A3* reduces proliferation, and the build-up of intracellular lactate is associated with enhanced production of reactive oxygen species [13, 25, 80]. This disrupted redox balance has been proposed to hamper oxidative phosphorylation, a metabolic pathway which is thought to be essential for cancer cell survival at low pHe [61]. A third explanation relates to intracellular accumulation of non-lactate monocarboxylates, such as pyruvate, when MCT activity is impaired. Indeed, MCT1 inhibition in glycolytic breast cancer cells co-expressing MCT1 and MCT4 acutely reduced pyruvate export, without a reduction in lactate export [41]. When these cells were xenografted into mice, MCT1 inhibition blocked the growth of mammary fat pad tumours.

Despite strong in vitro and clinical evidence for MCT essentiality, it remains unclear whether MCT inhibitors will be effective in the clinic. Firstly, acute reductions in MCT permeability by pharmacological inhibitors can be somewhat overcome over longer time periods by an increased [lactate] driving force, a phenomenon known as autoregulation [12]. Secondly, there are questions regarding the specificity of MCT essentiality to cancer. Indeed, MCT1 is expressed in almost all cell types and can act bi-directionally depending on the substrate gradient. In cancer, where fermentive rate is elevated, the gradient typically favours H^+^/lactate export [74]. However, the gradient can be reversed in many healthy tissues, facilitating lactate import. In the brain, lactate is one of the most crucial energy substrates after glucose [2]. Accordingly, MCT1 expression is highly localised around axons and oligodendrocytes [2]. Moreover, neurological sequelae have been described in patients with germline inactivating *SLC16A1* mutations [2]. This might raise concerns regarding the safety profile of the systemic administration of MCT1 inhibitors to cancer patients. Indeed, the oral MCT1 inhibitor, AZD3965, has reached a Phase 1 clinical trial, where adverse effects including retinopathy, fatigue, and systemic acidosis were observed [37].

## To what degree is there redundancy in the acid-base transport system of cancers?

A major biological phenomenon acting against gene essentiality is functional redundancy in systems, including pHi control. This considers whether the impairment to one acid-base transporter can be compensated for by another cell- or population-level mechanism. Functional redundancy among pHi regulatory systems would confer cancer cells with greater resistance to the therapeutic manipulation of acid-base balance. Moreover, it is well-recognised that functional redundancy is more likely if a gene has multiple paralogs with high sequence similarities [26], which is the case for many ABT-SLCs [3]. However, numerous in vitro studies have indicated that functional redundancy among ABT-SLCs may not always manifest. SiRNA-mediated knock-down of the acid-loading AE *SLC4A2* can raise steady-state pHi in colorectal cancer cells [60]. Even though there are far more types of acid-extruders than acid-loaders, piecewise knock-down of acid-extruders (*SLC9A1*, *SLC4A7*, or *SLC16A3*) can be sufficient to impair pHi control in cancer cells [4].

To explore whether these in vitro observations might translate to patients, we analysed the number of somatic mutations that tumours accrue in each sub-group of ABT-SLCs using the pan-cancer simple nucleotide variation data previously downloaded (Fig. 3f). For tumour samples carrying a single mutation to either an MCT, NHE, NBC, or AE transporter, we calculated the average number of additional somatic mutations within the affected transporter sub-group per tumour sample. Strikingly, when tumours carried a somatic mutation in an ABT-SLC, there were less than 0.5 additional somatic mutations/tumour sample affecting that same transporter sub-group. Whilst 17.58% of tumour samples carry at least one ABT-SLC mutation (Fig. 1b), few tumours are able to carry multiple somatic mutations affecting the same sub-group of ABT-SLC (i.e. MCT, NHE, NBC, or AE). Assuming that at least some somatic mutations affect protein function, this finding implies that there is a degree of functional redundancy within each sub-group of ABT-SLC that can compensate for a single somatic mutation, but less so for multiple mutated transporters. Relative to other ABT-SLCs, tumours carried significantly fewer additional MCT mutations (Fig. 3f), possibly related to the postulated essentiality of MCTs.

Whilst many NHEs, NBCs, and AEs do not exhibit functional redundancy in vitro [4], it is somewhat surprising that MCTs are the only ABT-SLCs that have been proposed as essential genes in human cancers thus far [7, 90]. A possible explanation for the disparity between observations in vitro and in patients is the nature of cell monocultures. The expression profile of a cancer cell line monoculture is almost infinitely narrower than the transcriptomic landscape of the genetically heterogenous cancer cell population within a tumour, where there is a much higher likelihood of paralog co-expression. This phenomenon is illustrated by studies of MCT inhibition in the lymphoblast-like cell line Raji, which express MCT1, but not MCT4 [25]. MCT1 inhibition by AZD3965 impairs Raji cell growth. However, in viral-driven lymphoma cell lines where MCT1 and MCT4 are co-expressed, neither AZD3965 nor the MCT4 inhibitor VB124 alone affect cell growth [13]. Only dual inhibition of both MCT isoforms is sufficient to impact proliferation. Clearly, functional redundancy is not always present in vitro due to cancer cell line-specific isoform expression that is unrepresentative of in vivo expression.

Compensation for a deficit in a cell’s pH-regulatory apparatus occurs not only at the level of an individual cell, but also at a population level. In population-level compensation, the ‘unit’ under consideration is a syncytial network of cancer cells coupled by gap junctions [75]. Gap junctions mediate the exchange and sharing of small metabolites between cancer cells [29, 30, 62, 75]. Whilst protons permeate gap junctions slowly due to their heavy cytoplasmic buffering, the ABT-SLC substrates lactate and bicarbonate are more rapidly dissipated, e.g. between pancreatic ductal adenocarcinoma cell lines via connexin43-based gap junctions [30]. In a heterogenous cancer cell population, defective pH regulation in one subclone might therefore be compensated for by a fully operational pH-regulatory apparatus in diffusively coupled neighbouring cells. For example, co-culture of *SLC9A1*^-/-^ and *SLC9A1* wildtype colorectal cancer cells rescues the defective pHi recovery of the *SLC9A1*^-/-^ cells following an acid load, likely due to metabolite dissipation via connexin26-based gap junctions [62]. Such diffusive coupling via gap junctions may permit population-level functional redundancy in ABT-SLCs in patients that cannot be observed in genetically homogenous cell line monocultures.

## Future directions

Physiologists will be familiar with Claude Bernard’s assertion that ‘the stability of the *milieu intérieur* is a condition for a free and independent life’ because it introduced the concept of homeostasis [40]. A homeostatic challenge at the cellular level is the control of solute and ion concentrations, which is why physiologists concur that SLCs are critical. Low pHe is a major homeostatic challenge facing cancer cells and exerts a substantial selection pressure in the context of intra-tumoural genetic and epigenetic heterogeneity [11]. Consequently, we sought to evaluate the role of ABT-SLCs in intra-tumoural evolution.

In many cancer types, we find that the proportion of tumours carrying somatic ABT-SLC mutations is proportional to tumour mutation burden. However, in UCEC, somatic ABT-SLC mutations are more prevalent than expected from the median tumour mutation burden (Fig. 2a). Furthermore, the majority of these mutations are predicted to detrimentally impact protein function (Fig. 3a–c). However, the role and mechanisms of pHi regulation specific to the endometrium remain largely unexplored. Such investigations are warranted, not least due to the unique physiology of the endometrium. Physiological hypoxia has been proposed to occur in the endometrium during menses [58, 68]. Hypoxia-inducible factor (HIF) signalling augments lactate/H^+^ production via upregulation of glycolytic enzymes, and MCT4 is a known HIF-1 target [82]. Whilst the average age of the TCGA-UCEC cohort is 63.9±11.1 years (mean±standard deviation) [86], it could be interesting to explore whether alterations in endometrial ABT-SLC function earlier in life impact tumourigenesis.

In pan-cancer analyses, we find that *SLC4A3* ranks as the second most commonly mutated ABT-SLC (Fig. 1b). In UCEC, *SLC4A3* can be highly expressed at transcript level relative to other ABT-SLCs (Fig. 2c). *SLC4A3* somatic mutation is associated with significantly lower *SLC4A3* mRNA levels (Fig. 3d), and most *SLC4A3* missense mutations are predicted to be detrimental to SLC4A3 function (Fig. 3a–c). However, widely utilised algorithm-based approaches to detect cancer driver genes do not definitively identify *SLC4A3* when considering both *p*-value *and* fdr (Fig. 2d, e). Moreover, it is surprising that many ABT-SLCs are not identified by contemporary in silico studies of selection in cancer [6, 28, 56], given that germline ABT-SLC SNPs can increase cancer risk [20, 95] and genetic ablation of ABT-SLCs substantially impairs cancer cell fitness in vitro [4, 60].

This paradox can be explained in terms of the non-stoichiometric relationship between genotype and phenotype and draws caution to our heavy reliance on genomics in oncology. Intra-tumoural evolution, like species-level evolution, selects for phenotype rather than genotype *per say* [11]. Phenotype is influenced at a myriad of levels: not only by genomics and epigenomics, but also by factors such as post-translational modification, neighbouring cell function, allosteric regulation, and a cell’s chemical microenvironment. There is clearly a need to explore ABT-SLCs beyond cancer cell line monocultures towards informative studies in human cancers. A mutation-based approach is currently one of the few feasible methods to explore SLCs in human tumour evolution, yet it is important to acknowledge its reductionist nature in comparison to the measurement of phenotype, i.e. SLC-generated ionic or solute fluxes.

A clear direction of future work is thus to annotate important physiological parameters into analyses of ABT-SLCs in patients. Microenvironmental factors which are intrinsically linked to ABT-SLC function, such as pHe and hypoxia, could be integrated into analyses that preserve the location of cells within the microenvironment of a human tumour. These include spatially resolved genomic and transcriptomic sequencing, such as slide-DNA-seq [93] or in situ genome sequencing [67]. Surface membrane-expressed markers of hypoxia (such as CA9 [43]) or acid-adaptation (such as LAMP2 [60]) might be leveraged for this purpose. For example, spatial transcriptomics platforms could be combined with immunofluorescence staining of markers [36], or markers could be used for cell sorting followed by single-cell exome sequencing [78]. Given that robust intracellular pH-reporters, such as cSNARF-1, already exist, ABT-SLC flux could be measured directly in patient-derived organoids and xenografts. Physiologically and clinically relevant methods to interrogate ABT-SLCs in cancer will ultimately yield improved therapeutic targets.

Our findings are consistent with previous reports that MCTs are essential in cancer [7, 90]. Pan-cancer, *SLC16A3* and *SLC16A8* are somatically mutated in less than 1% of tumours (Fig. 1b). When ABT-SLCs are ranked by the abundance of somatic mutations pan-cancer, the cumulative incidence of somatic MCT mutations is even below that of SLC transporters which supply cancer cells with critical macromolecules for cell division, including glucose, amino acids, and nucleotides/nucleosides (Fig. 1c). Analysis of tumour samples carrying multiple somatic mutations within each ABT-SLC sub-group suggests that there is less functional redundancy within MCTs relative to AEs, NBCs, and NHEs (Fig. 3f). Moreover, UCEC progression is significantly hampered in tumours containing somatic MCT mutations (Fig. 3e). Our findings support efforts to develop MCT inhibitors for clinical use [37]; however, future work might focus on improving selectivity.

MCTs, and indeed other ABT-SLCs, are expressed in many non-tumour tissues due to their near-universal housekeeping functions and roles in systems-level physiological processes [74]. Targeting any ABT-SLC therefore risks adverse effects. A potential solution lies in novel therapeutic delivery systems. Given the close relationship between ABT-SLCs and intra-tumoural acidosis, pHe-dependence of therapeutic delivery will be critical to improving selectivity. For example, cargo unloading of emerging delivery mechanisms, such as extracellular vesicles, could be targeted to acid-induced epitopes on the cell surface [49]. pH-(low) insertion peptides (pHLIP) are an exciting technology based on peptide constructs which can fold into a transmembrane helix, allowing insertion and crossing of the cell membrane, only at low pHe [54]. pHLIP constructs may be engineered to deliver cargo into tumour cells in vivo, such as fluorescent markers or even therapeutics. Indeed, a Phase 2a clinical trial employing imaging of fluorescently labelled pHLIP to guide tumour margin detection in breast cancer surgery has recently begun (ClinicalTrials.gov Identifier: NCT05130801).

Ultimately, genomic approaches alone are insufficient to elucidate and clinically harness acid-base transport in cancer. Clearly, physiology-based approaches are necessary in both target validation and delivery mechanisms to enable ABT-SLCs to become effective therapeutic targets in oncology.

### Supplementary information

ESM 1 (PDF 40 kb)

## Data Availability

The datasets analysed during the current study are available from the National Cancer Institute GDC Data Portal, https://portal.gdc.cancer.gov/.
