# Supplementary material for: What can we learn about acid-base transporters in cancer from studying somatic mutations in their genes?
Source: Pflugers Arch. 2023 Nov 24;476(4):673–88. doi: 10.1007/s00424-023-02876-y (PMC11006749; doi:10.1007/s00424-023-02876-y)
Supplement: Supplementary file 1 — (PDF 40 kb) [file 424_2023_2876_MOESM1_ESM.pdf]

TCGA study abbreviation	TCGA study data plotted in Fig 2a.	Labelled in Fig. 2a
**SKCM**	Skin Cutaneous Melanoma	Yes
**LUSC**	Lung squamous cell carcinoma	Yes
**LUAD**	Lung adenocarcinoma	Yes
**BLCA**	Bladder Urothelial Carcinoma	Yes
**UCEC**	Uterine Corpus Endometrial Carcinoma	Yes
**COAD**	Colon adenocarcinoma	Yes
**STAD**	Stomach adenocarcinoma	Yes
**CESC**	Cervical squamous cell carcinoma and endocervical adenocarcinoma	Yes
**READ**	Rectum adenocarcinoma	Yes
**HNSC**	Head and Neck squamous cell carcinoma	Yes
**LIHC**	Liver hepatocellular carcinoma	Yes
**DLBC**	Lymphoid Neoplasm Diffuse Large B-cell Lymphoma	Yes
**ESCA**	Esophageal carcinoma	Yes
**OV**	Ovarian serous cystadenocarcinoma	Yes
**BRCA**	Breast invasive carcinoma	Yes
**GBM**	Glioblastoma multiforme	Yes
**KICH**	Kidney Chromophobe	Yes
**ACC**	Adrenocortical carcinoma	Yes
**UVM**	Uveal Melanoma	Yes
**THCA**	Thyroid carcinoma	Yes
**UCS**	Uterine Carcinosarcoma	Yes
**KIRC**	Kidney renal clear cell carcinoma	Yes
**KIRP**	Kidney renal papillary cell carcinoma	Yes
**SARC**	Sarcoma	Yes
**CHOL**	Cholangiocarcinoma	Yes
**PAAD**	Pancreatic adenocarcinoma	No
**MESO**	Mesothelioma	No
**PRAD**	Prostate adenocarcinoma	No
**LGG**	Brain Lower Grade Glioma	No
**TGCT**	Testicular Germ Cell Tumors	No
**THYM**	Thymoma	No
**LAML**	Acute Myeloid Leukemia	No
**PCPG**	Pheochromocytoma and Paraganglioma	No
